# Circular RNA circ-CCAC1 Facilitates Adrenocortical Carcinoma Cell Proliferation, Migration, and Invasion through Regulating the miR-514a-5p/C22orf46 Axis

**DOI:** 10.1155/2020/3501451

**Published:** 2020-10-26

**Authors:** Wei Li, Rengong Liu, Dongmei Wei, Wei Zhang, Heyan Zhang, Wenjun Huang, Liguo Hao

**Affiliations:** ^1^Department of Endocrinology, The First Hospital of Qiqihar, Qiqihar 161000, China; ^2^Department of Urinary Surgery, The First Hospital of Qiqihar, Qiqihar 161000, China; ^3^Department of Science and Education, The First Hospital of Qiqihar, Qiqihar 161000, China; ^4^Department of Molecular Imaging, Qiqihar Medical University, Qiqihar 161000, China

## Abstract

Adrenocortical carcinoma (ACC) is a rare but clinically aggressive endocrine malignancy. Circular RNAs (circRNAs) were found to play key roles in tumorigenesis. In the current study, we aimed to investigate the functions and mechanisms of a novel circRNA, circ-CCAC1, in ACC cells. circ-CCAC1 expression levels in ACC tissue specimens and cell lines were evaluated by RT-qPCR. Kaplan-Meier analysis was applied to explore the relationship between circ-CCAC1 and patients' prognosis. Cell counting kit-8 (CCK-8), colony formation, acridine orange/ethidium bromide (AO/EB) double fluorescence staining, and Transwell assays were performed to evaluate the functions of circ-CCAC1 in ACC cells. Bioinformatics analysis and a dual-luciferase reporter assay were utilized to explore the mechanisms of circ-CCAC1. As a result, circ-CCAC1 was overexpressed in ACC tissue samples and cell lines and correlated with poor prognosis. Gain- and loss-of-function tests demonstrated that circ-CCAC1 acted as an oncogene in ACC. What is more, circ-CCAC1 enhanced C22orf46 expression by sponging miR-514a-5p in ACC cells. A rescue assay illustrated that circ-CCAC1 facilitated ACC progression through miR-514a-5p/C22orf46 signaling. To sum up, we identified a novel circRNA, circ-CCAC1, which may be used as a potential therapeutic target for ACC.

## 1. Introduction

Adrenocortical carcinoma (ACC) is a rare but clinically aggressive endocrine malignancy [1–3]. The incidence of ACC is around 0.7/10000 to 2/10000. ACC frequently occurs in adolescent [4, 5]. Although the treatment of ACC has made remarkable progression, the prognosis of ACC is still unfavorable due to local infiltration or extensive metastasis [6]. Moreover, ACC shows medium-low response rates to conventional chemotherapy and emergence of chemoresistance properties [7]. Therefore, understanding the molecular regulation mechanism of ACC progression and identifying an effective therapeutic target are the key issues for the treatment of ACC.

Previous research used whole genome sequencing, and findings indicated that greater than 98% of a genome was transcribed into noncoding RNAs (ncRNAs). Circular RNAs (circRNAs) are a novel type of ncRNAs. However, emerging evidence indicates that several circRNAs are able to encode peptides [8]. For instance, circ-SHPRH encodes a novel protein, SHPRH-146aa [9]. circRNAs are characterized by a closed-loop structure with limited protein coding capacity [10, 11] and have been found to function as oncogenes or cancer suppressor genes in a wide variety of human cancers [12, 13]. For example, circ_0001649 inhibits cholangiocarcinoma progression *in vitro* and *in vivo* [14]. Accumulating evidence has indicated that circRNAs play important roles in cancer with respect to cell proliferation, migration, invasion, and apoptosis, as well as can be important biomarkers that can predict patients' prognosis [15, 16]. Furthermore, important interactions between mammalian circRNA and miRNA have been identified in assessments of various types of cancers [17]. For example, hsa_circ_101141 acts as a competing endogenous RNA (ceRNA) to facilitate progression of hepatocellular carcinoma by regulating miR-1297/ROCK1 signaling [18]. Previously, the researchers identified a novel circRNA, circ-CCAC1, which functions as an oncogene in cholangiocarcinoma [19]. circ-CCAC1 (circBase ID: hsa_circ_0043469; circBank ID: hsa_circERBB2_014) is located on chr17: 37880978-37882106 and looped by exons 23-26 of ERBB2. The spliced variant of circ-CCAC1 is 565 nucleotides long. Interestingly, we found circ-CCAC1 was also remarkably increased in ACC, which is related to poor prognosis among ACC patients. Silencing of circ-CCAC1 inhibits ACC cell progression *in vitro*. Mechanistically, circ-CCAC1 is the sponge of miR-514a-5p, which promotes C22orf46 expression, thus promoting the proliferation and aggressiveness of ACC. Our findings provided a new insight for exploring a potential therapeutic strategy for the treatment of ACC.

## 2. Materials and Methods

### 2.1. Patients

Starting from January 2014 and proceeding through February 2016, we collected 48 paired samples of ACC tissues and corresponding noncancerous tissues from patients admitted to the First Hospital of Qiqihar by way of surgical resection (Table S1). All pathological specimens were independently diagnosed by three clinical pathologists. After sectioning, all ACC tissues and noncancerous tissues were immediately stored in liquid nitrogen and then preserved in a -80°C freezer. All aspects of experimental designs and protocols were reviewed and approved by the Committee for the Protection of Human Subjects at the First Hospital of Qiqihar. All patients signed written informed consent forms before surgical resection.

### 2.2. Cell Lines and Transfection

Human ACC cell lines (H295R and SW-13) and an immortalized normal cell line (Y1) were acquired from the Cell Bank of the Chinese Academy of Sciences (Shanghai, China). We cultured all the cell lines in RPMI-1640 medium following the manufacturer's protocols and supplemented the medium with 10% fetal bovine serum (FBS, Gibco). Next, we incubated all samples in a humidified atmosphere with a constant temperature of 37°C and a 5% CO_2_ atmosphere.

circ-CCAC1-specific small interfering RNA (si-circ-CCAC1-1, si-circ-CCAC1-2), C22orf46-specific siRNA (si-C22orf46), siRNA-negative control (si-NC), miR-514a-5p mimics, inhibitor, and negative controls were obtained from GenePharma Co. Ltd. (Shanghai, China). Lipofectamine™ 3000 (Invitrogen, USA) was used to transfect samples with si-RNA or miRNAs. 125 *μ*L serum-free medium was used to dilute 5 *μ*L Lipofectamine™ 3000 in a 1.5 mL EP tube. Meanwhile, 5 *μ*L siRNA (20 *μ*M) or 2.5 *μ*g plasmid vector along with 5 *μ*L P3000™ reagent was diluted in 125 *μ*L serum-free medium. After 5 minutes of incubation at room temperature, the reagents in two tubes were combined. 15-20 minutes later, the mixtures were added into a 2.5 cm dish filled with serum-free medium. After 8 hours of incubation, the medium was replaced by the medium containing 10% FBS. All transfection experiments were repeated three times. The knockdown efficiency was confirmed by real-time quantitative PCR (RT-qPCR) analysis. The targeted sequences of the siRNAs specifically targeting to circ-CCAC1 are listed as following: si-circ-CCAC1-1, 5′-ATGGTCAAATGAAGCATACGT-3′, and si-circ-CCAC1-2, 5′-GATCATGGTCAAATGAAGCAT-3′.

### 2.3. RT-qPCR

We used TRIzol (Thermo Scientific, USA) to isolate total RNA from samples followed by the manufacturer's protocols. Next, we quantified measures of total RNA using a NanoDrop 2000. We next reverse-transcribed fragments in samples into resultant cDNA. We used an ABI 7500 Fast RT-qPCR System for RT-qPCR assessments and used 20 *μ*L for each RT-qPCR reaction mixture. Relative expression levels were calculated by using the 2^−*ΔΔ*Ct^ method. PCR primers are listed as follows: circ-CCAC1: 5′-TGTGGAGTTATGGTGTGACTGT-3′ (forward) and 5′-GCCATCACGTATGCTTCATTTG-3′ (reverse). GAPDH: 5′-GGGAGCCAAAAGGGTCAT-3′ (forward) and 5′-GAGTCCTTCCACGATACCAA-3′ (reverse).

Nuclear and cytoplasmic fractions of H295R/SW-13 cells were partitioned using a PARIS Kit (Thermo Scientific). A total of 10^7^ fresh cultured cells were collected, placed on ice, and resuspended with 500 *μ*L ice-cold cell fractionation buffer. Cells were incubated on ice for 15 min. Samples were centrifuged at 500 g for 5 min, and then the cytoplasmic fraction was carefully aspirated away to a new tube from the nuclear pellets. RNA isolation from chromatin and nucleoplasm was performed using a TRIzol reagent (Invitrogen) according to the manufacturer's protocol.

### 2.4. Western Blotting

The cultured cells were lysed in 2% SDS buffer plus protease and phosphatase inhibitor cocktails (Thermo Scientific, USA). BCA protein assay (Solarbio) was used to measure the protein concentrations. Equivalent samples were separated by SDS-PAGE and then transferred into a PVDF membrane (Millipore). After immersion in 5% skim milk at 22-25°C, the membrane was incubated with primary antibodies overnight at 4°C. After incubation with the HRP-conjugated secondary antibody for 2 h, the membrane was visualized using an ECL kit (Beyotime).

### 2.5. Dual-Luciferase Reporter Gene Test

starBase 2.0 and circBank databases were utilized for predicting the miRNAs potentially interacted with circ-TOP2A. The binding relationship of 3′-untranslated region (UTR) of C22orf46 and miR-514a-5p was predicted by the TargetScan database. For the dual-luciferase reporter gene test, we seeded ACC cells into a 24-well plate and cultured the plate for 24 h. To confirm the interaction between miR-514a-5p and circ-CCAC1/3'-UTR of C22orf46, we transfected the following into ACC cells: circ-CCAC1/3′-UTR of C22orf46 wild reporter vector (pmirGLO), circ-CCAC1/3′-UTR of C22orf46 mutant reporter vector (Mut), and miR-514a-5p mimics or miR-NC. The Dual-Luciferase Reporter Assay System (Promega, USA) was used to examine measures of luciferase intensity.

### 2.6. Cell Counting Kit-8 (CCK-8)

The viability of transfected ACC cells was examined by CCK-8 assay. The cells were collected, and the concentration was adjusted to 1500 cells/well before they were maintained in a cell incubator for 0, 24, 48, 72, and 96 hours. After the indicated specific treatments, 10 *μ*L of CCK-8 was supplied to the wells and maintained at 37°C. After incubation for additional 2 h, a microplate reader (Multiskan EX, LabSystems, Helsinki, Finland) was used to measure the absorbance at 450 nm.

### 2.7. Colony Forming Test

Transfected ACC cells were seeded in 6-well plates with a total of 500 cells per well with medium containing 10% FBS and incubated at 37°C. After 10 days of incubation, the colonies were fixed with 4% paraformaldehyde, then incubated with crystal violet (Sigma-Aldrich, Shanghai, China) for another 15 minutes and enumerated under the microscope.

### 2.8. Apoptosis Detection

For the AO/EB experiment, tumor cells were preseeded and cultured overnight in a 6-well dish. The preconfigured AO/EB solution (Solarbio, Beijing, China) was added to each well. Finally, the apoptosis level was observed under a fluorescent microscope system (Leica, Buffalo Grove, IL, USA). Due to the different transmembrane characteristics of AO/EB, normal and apoptotic cells could be identified. AO dyestuff could penetrate intact cell membranes and specifically bind to nuclear DNA, emitting bright green fluorescence. Instead, EB dyestuff could only enter damaged cell, emitting red fluorescence.

### 2.9. Transwell Assay

Matrigel was dropwise added into Transwell upper chambers with routine protocols. The chambers were kept in a 4°C air-dried condition. ACC cells were cultured till the phase of logarithmic growth. Cells were harvested with a diluted cell density of 1 × 10^6^. We then added 200 *μ*L of cell suspension into the Transwell upper chamber. Meanwhile, 600 *μ*L fresh nutrient medium was added into the Transwell lower chamber. The remaining Matrigel and ACC cells were cleaned after 24 h culture. Crystal violet staining (30 minutes) was performed for cells in the Transwell lower chamber.

### 2.10. Data Analysis

SPSS 22.0 and GraphPad Prism 8.3.0 were used for statistical analysis. The experimental data were all presented as the mean ± standard deviation (S.D.). The survival curve was generated and analyzed via Kaplan-Meier plot and log-rank test. To compare the difference of groups, we used Student's *t*-test and one-way analysis of variance (ANOVA). *P* < 0.05 indicated the presence of a significant difference.

## 3. Results

### 3.1. circ-CCAC1 Is Upregulated in ACC and Correlates with Poor Prognosis

**Figure 1:**
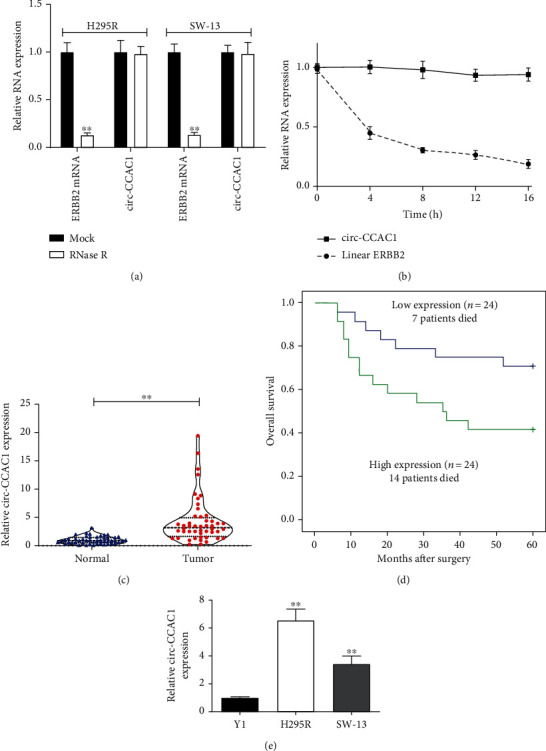
circ-CCAC1 expression in ACC tissues and cells and its clinical importance. (a) circ-CCAC1 was resistant to RNase R digestion in ACC cells. (b) Relative circ-CCAC1 and linear ERBB2 mRNA expression at different time points. (c) circ-CCAC1 and ERBB2 mRNA expression in 48 pairs of ACC tissues/adjacent normal tissues by RT-qPCR. (d) Kaplan-Meier analysis with log-rank test for overall survival in ACC patients according to circ-CCAC1 expression. (e) Relative expression of circ-CCAC1 in ACC and normal cells by RT-qPCR. ^∗∗^*P* < 0.01.

circ-CCAC1 was more stable than ERBB2 mRNA under the circumstance of RNase R (Figure 1(a)). Total RNA was isolated to measure the expression levels of circ-CCAC1 and ERBB2 mRNA after treatment with actinomycin D at different time points. We found that the half-life of circ-CCAC1 was longer than its linear isoform (ERBB2 mRNA) (Figure 1(b)). circ-CCAC1 expression was significantly higher in ACC tissues compared to noncancerous samples (Figure 1(c)). According to the median value of circ-CCAC1, we classified the enrolled ACC patients into two groups (high and low expression groups). We found that high circ-CCAC1 expression was linked to worse overall survival (*P* = 0.006) for the ACC patients after surgery (Figure 1(d)). Next, we evaluated the expression of circ-CCAC1 in ACC cell lines (H295R and SW-13) and a normal cell line (Y1). As a result, the two ACC cell lines exhibited significantly higher levels of circ-CCAC1 compared to Y1 cells (Figure 1(e)).

### 3.2. circ-CCAC1 Promotes ACC Cell Proliferation and Aggressiveness

**Figure 2:**
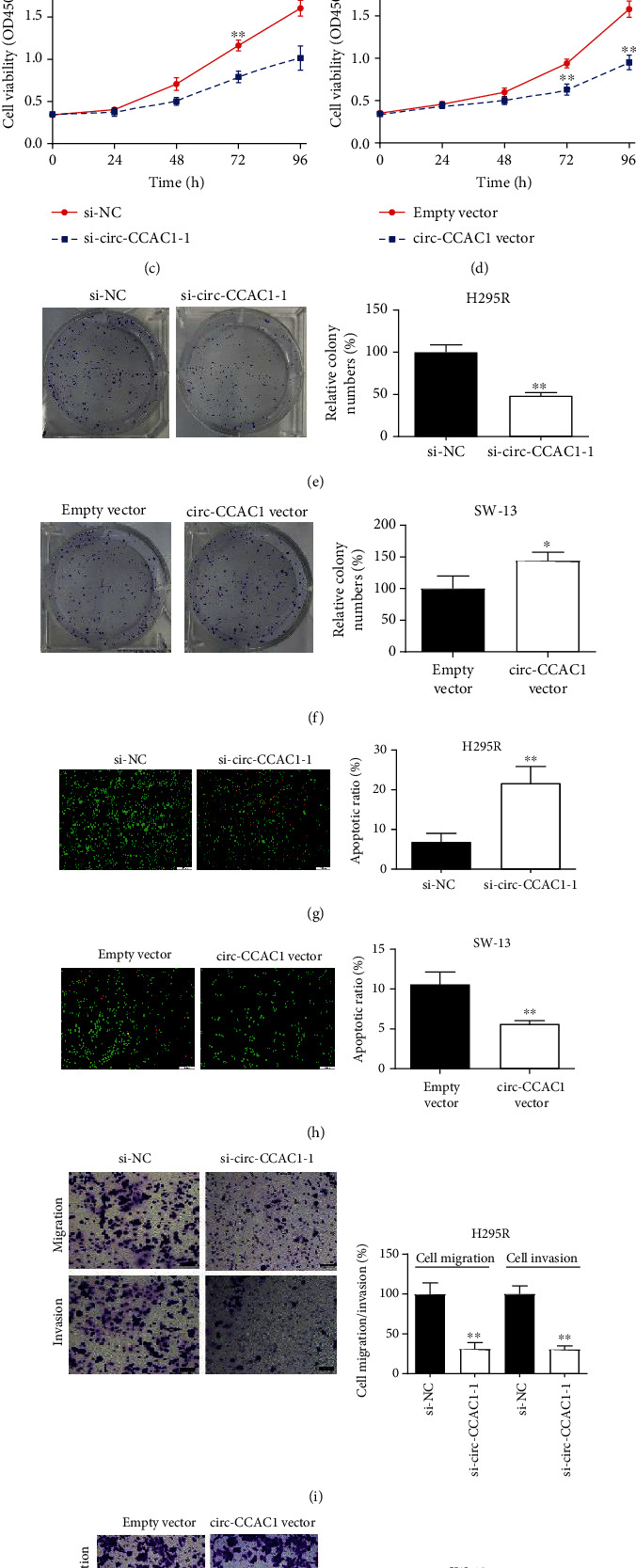
circ-CCAC1 facilitates ACC cell progression. (a) Relative expression of circ-CCAC1 was detected by RT-qPCR after transfection. (b) Relative expression of ERBB2 mRNA was detected by RT-qPCR after transfection. (c) Cell viability was detected after downregulating circ-CCAC1 in H295R cells by CCK-8. (d) Cell viability was detected after upregulating circ-CCAC1 in SW-13 cells by CCK-8. (e) Clone-forming ability was detected after downregulating circ-CCAC1 in H295R cells by colony formation assay. (f) Clone-forming ability was detected after upregulating circ-CCAC1 in SW-13 cells by colony formation assay. (g) Cell apoptosis was detected after downregulating circ-CCAC1 in H295R cells by AO/EB staining assay. (h) Cell apoptosis was detected after upregulating circ-CCAC1 in SW-13 cells by AO/EB staining assay. (i) Cell migration and invasion was detected after downregulating circ-CCAC1 in H295R cells by Transwell assay. (j) Cell migration and invasion were detected after upregulating circ-CCAC1 in SW-13 cells by Transwell assay. ^∗^*P* < 0.05, ^∗∗^*P* < 0.01.

To facilitate explorations of the roles that circ-CCAC1 may have played in the progression of ACC, we designed two circ-CCAC1 siRNAs that would knock down circ-CCAC1. We found that the levels of circ-CCAC1 were notably downregulated in H295R cells that had been transfected with si-circ-CCAC1-1/-2 (Figure 2(a)). Additionally, an overexpression study was conducted on SW-13 cells due to its lowest expression of circ-CCAC1, and the overexpression efficiency of circ-CCAC1 vector was favorable (Figure 2(a)). Furthermore, ERBB2 mRNA expression levels were unaffected after circ-CCAC1 knockdown/overexpression (Figure 2(b)). We used CCK-8 and colony formation tests to detect ACC cell viability and clone-forming capacity affected by circ-CCAC1. The results confirmed that silencing of circ-CCAC1 significantly inhibited cell viability and clone-forming capacity (Figures 2(c) and 2(e)). Conversely, overexpression of circ-CCAC1 accelerated cell growth *in vitro* (Figures 2(d) and 2(f)). Furthermore, AO/EB double staining assay indicated that the cell growth-promoting role of circ-CCAC1 is partly attributed to its suppression on cell apoptosis in ACC cells (Figures 2(g) and 2(h)). Results from Transwell assays indicated that silencing of circ-CCAC1 attenuated the migratory and invasive potential of H295R cells (Figure 2(i)). By contrast, elevated circ-CCAC1 strengthened the migratory and invasive capacities of SW-13 cells (Figure 2(j)).

### 3.3. circ-CCAC1 Enhanced C22orf46 Expression by Sponging miR-514a-5p in ACC

**Figure 3:**
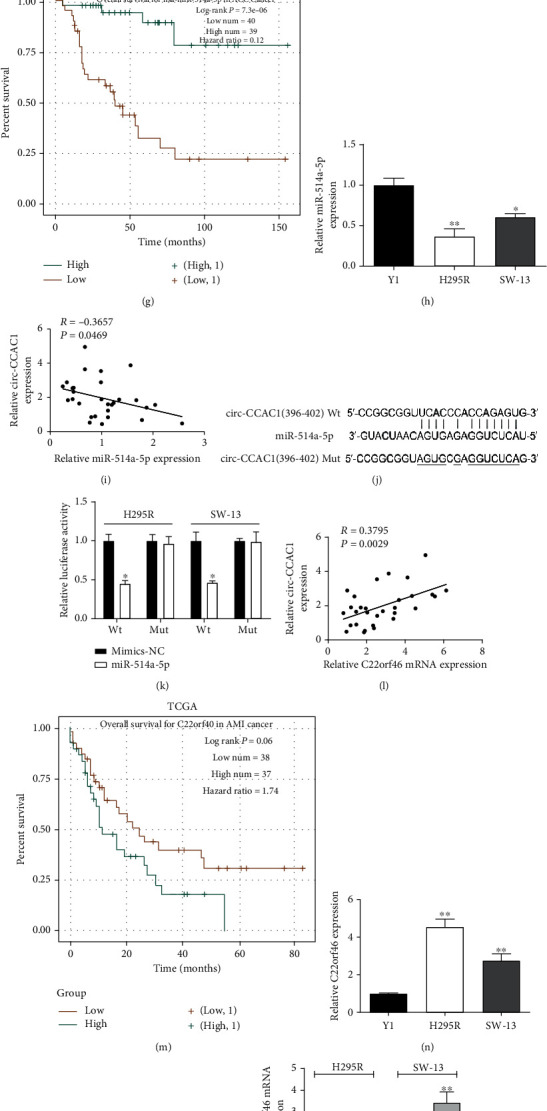
circ-CCAC1 sponges miR-514a-5p to upregulate C22orf46 expression in ACC. (a) RT-qPCR detection of the percentage of circ-CCAC1 in the cytoplasmic and nuclear fractions of H295R cells. (b) RT-qPCR detection of the percentage of circ-CCAC1 in the cytoplasmic and nuclear fractions of SW-13 cells. (c) Venn diagram showing the number of overlapping miRNAs. (d) Relative expression levels of miR-182-5p, miR-1343-3p, miR-514a-5p, miR-3619-5p, and miR-6746-5p were detected after silencing of circ-CCAC1 in H295R cells. (e) Relative expression levels of miR-182-5p, miR-1343-3p, miR-514a-5p, miR-3619-5p, and miR-6746-5p were detected after silencing of circ-CCAC1 in SW-13 cells. (f) Relative expression of miR-514a-5p in ACC tissues and normal tissues by RT-qPCR. (g) Kaplan-Meier analysis of overall survival in ACC patients according to miR-514a-5p expression by TCGA data. (h) Relative expression of miR-514a-5p in ACC and normal cells by RT-qPCR. (i) Correlation analysis of circ-CCAC1 and miR-514a-5p expression in ACC samples. (j) Schematic illustration of circ-CCAC1-Wt/Mut luciferase reporter vectors. (k) The binding ability between circ-CCAC1 and miR-514a-5p was detected by dual-luciferase reporter assay in H295R and SW-13 cells. (l) Correlation analysis of circ-CCAC1 and C22orf46 mRNA expression in ACC samples. (m) Kaplan-Meier analysis of overall survival in ACC patients according to C22orf46 expression by TCGA data. (n) Relative expression of C22orf46 mRNA in ACC and normal cells by RT-qPCR. (o) Relative expression of C22orf46 in ACC and normal cells by Western blotting. (p) C22orf46 mRNA expression was detected after down-/upregulating miR-514a-5p in H295R and SW-13 cells by RT-qPCR. (q) Schematic illustration of C22orf46 3′-UTR-Wt/Mut luciferase reporter vectors. (r) The binding ability between C22orf46 3′-UTR and miR-514a-5p was detected by dual-luciferase reporter assay in H295R and SW-13 cells. ^∗^*P* < 0.05, ^∗∗^*P* < 0.01.

As displayed in Figures 3(a) and 3(b), circ-CCAC1 was mainly localized to the cytoplasm of the two ACC cell lines. circRNAs can affect tumor cell malignant behaviors via sponging certain microRNAs [17, 18]. Therefore, we hypothesized miRNAs would bind to circ-CCAC1 and examined this prediction by using web-based circRNA-miRNA prediction tools. As a result, five miRNAs (miR-182-5p, miR-1343-3p, miR-514a-5p, miR-3619-5p, and miR-6746-5p) were predicted by both circBank and starBase 2.0 databases (Figure 3(c)). Silencing of circ-CCAC1 increased miR-514a-5p expression in H295R and SW-13 cells. The expression levels of other miRNAs were unchanged (Figures 3(d) and 3(e)). In addition, miR-514a-5p expression was downregulated in ACC samples relative to their normal counterparts (Figure 3(f)). TCGA datasets indicated that the patients with low expression of miR-514a-5p had a worse prognosis (Figure 3(g)). Furthermore, miR-514a-5p was downregulated in H295R and SW-13 cells than Y1 cells (Figure 3(h)). A negative association between circ-CCAC1 and miR-514a-5p was identified in ACC tissues (Figure 3(i)). We constructed a luciferase reporter vector of Wt and Mut circ-CCAC1 (Figure 3(j)). The vectors were cotransfected with miR-514a-5p mimics or mimics-NC in H295R and SW-13 cells. We found that miR-514a-5p mimics remarkably suppressed the luciferase signal relative to the negative control (Figure 3(k)). The TargetScan database was used to predict the downstream targets of miR-514a-5p, and C22orf46 was chosen for further study. Pearson's correlation analysis demonstrated that circ-CCAC1 expression was positively correlated with the levels of expression of C22orf46 for our examinations of ACC human tissue samples (Figure 3(l)). TCGA datasets indicated that the patients with upregulated C22orf46 expression had a worse overall survival (Figure 3(m)). Next, we examined expression levels of C22orf46 and found significantly higher levels of C22orf46 in ACC cells (Figures 3(n) and 3(o)). Additionally, we found that knocking down of miR-514a-5p significantly enhanced the levels of C22orf46 mRNA in H295R cells, whereas ectopic-expressed miR-514a-5p attenuated C22orf46 mRNA expression (Figure 3(p)). Dual-luciferase reporter assays indicated that cotransfection with Wt-C22orf46 3′-UTR reporter and miR-514a-5p mimics induced significant inhibition of luciferase activity in ACC cells (Figures 3(q) and 3(r)).

### 3.4. circ-CCAC1 Plays an Oncogenic Role via Upregulating C22orf46 Expression in ACC Cells

**Figure 4:**
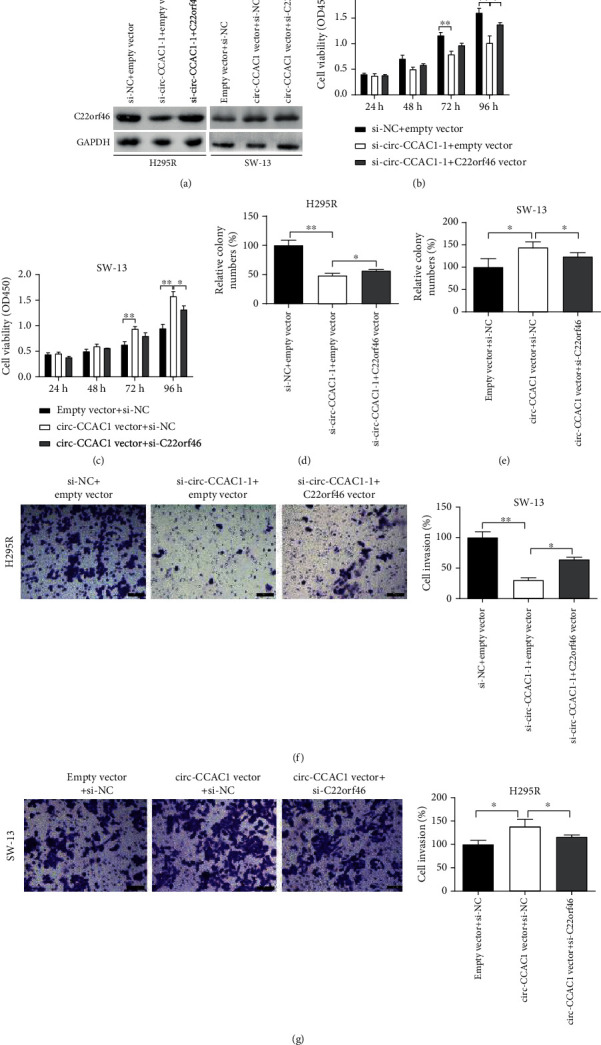
circ-CCAC1 facilitates ACC cell proliferation and aggressiveness via upregulating C22orf46 expression. (a) The protein level of C22orf46 was detected by Western blot after transfection in H295R and SW-13 cells. (b, c) CCK-8 assay was conducted to evaluate the viability of H295R and SW-13 cells after transfection. (d, e) Colony formation assay was conducted to evaluate the clone forming ability of H295R and SW-13 cells after transfection. (f, g) Transwell assay was conducted to evaluate the invasion of H295R and SW-13 cells after transfection. ^∗^*P* < 0.05, ^∗∗^*P* < 0.01.

We then cotransfected with si-circ-CCAC1-1 and C22orf46 vector in H295R cells, followed by Western blotting. circ-CCAC1 inhibition downregulated C22orf46 expression, whereas cotransfection with C22orf46 vector significantly increased C22orf46 expression levels (Figure 4(a)). In addition, ectopic expression of circ-CCAC1 enhanced C22orf46 expression in SW-13 cells. While, after cotransfection with si-C22orf46, the expression of C22orf46 was partially reversed (Figure 4(a)). CCK-8, colony formation, and Transwell experiments displayed that increasing C22orf46 reversed the inhibition of H295R cell growth and invasion caused by si-circ-CCAC1-1 (Figures 4(b), 4(d), and 4(f)). Overexpression of circ-CCAC1 triggered ACC cell progression, while this effect was partially reversed by cotransfection with si-C22orf46 (Figures 4(c), 4(e), and 4(g)).

## 4. Discussion

ACC is one of the most aggressive malignancies worldwide [2, 3]. Although remarkable progression has been achieved recently in the diagnosis and treatment of ACC, the prognosis is still dismal in ACC patients [4]. Although numerous studies have revealed that alterations in the oncogene and tumor suppressor gene contribute to the progression and metastasis of ACC [3], the precise molecular mechanism remains vague. Accumulating lines of evidence indicates that circRNAs play important roles in the dynamics underlying epigenetic regulation, transcription, and posttranscriptional regulation and may facilitate development and progression of tumorigenesis [16–18]. For example, circRNA GLIS2 promotes colorectal cancer cell motility via activation of the NF-*κ*B pathway [20]. A recent study indicated that circRNA_100782 promotes proliferation and metastasis of gastric cancer by downregulating tumor suppressor gene Rb by adsorbing miR-574-3p in a sponge form [21]. Mounting studies have shown that circRNAs can regulate the biological behaviors of cancer cells via target genes, thus exerting a pivotal role in the progression of malignancies [22]. Zhang et al. found that overexpressed circ-PIP5K1A contributes to colon cancer progression by suppressing miR-1273a expression [23]. The other study indicated that circ-TSPAN4 facilitates lung adenocarcinoma metastasis by elevating ZEB1 via sponging miR-665 [24]. However, the functions and mechanisms of circRNAs in ACC remain unclear. A recent study indicated that circ-CCAC1 was enhanced in cholangiocarcinoma and indicated that circ-CCAC1 was likely to affect tumorigenesis and metastasis in human cancers [19]. circ-CCAC1 is located on chr17: 37880978-37882106 and is looped by exons 23-26 of *ERBB2*. We found that circ-CCAC1 expression was also overexpressed in ACC tissue specimens than normal tissues and correlated with worse overall survival. Nevertheless, the independent prognostic role of circ-CCAC1 was not investigated. We then evaluated the functions of circ-CCAC1 in tumorigenesis and progression of ACC cells. Gain- and loss-of-function assays illustrated that circ-CCAC1 significantly increased the viability, clone-forming, migration, and invasion of ACC cells. What is more, the cell growth-promoting role of circ-CCAC1 was partially dependent on its suppression on cell apoptosis.

Increasing evidence showed that there were extensive interaction networks involving ceRNA, in which circRNAs could regulate the target RNA by binding to the miRNAs and titrating from the binding site on the protein coding messenger [25]. Target molecules regulated mutual expression by competing to bind miRNA's response element (MRE). Evidence suggested that the ceRNA regulatory model had been validated in other cancers [25, 26]. The localization of circRNAs suggests how they exert their functions. circ-CCAC1 primarily localized to the cytoplasm rather than the nucleus, suggesting its mechanism in the posttranscriptional level. In this work, our results indicated that miR-514a-5p expression was significantly lower in ACC tissues and cell lines. Meanwhile, a negative correlation was observed between miR-514a-5p and circ-CCAC1 expression. We found that circ-CCAC1 could sponge and negatively regulate miR-514a-5p expression in ACC cells. In fact, miR-514a-5p has been confirmed as a tumor suppressor gene in some types of human cancers [27]. Moreover, miR-514a-5p was regulated at the posttranscriptional level. For example, miR-514a-5p could be sponged by long noncoding RNA TRIM52-AS1 and SNHG7, thus releasing its suppression on MRPS18A and ELAVL1, respectively [28, 29]. However, the functions and mechanisms of miR-514a-5p in ACC are still unclear. The above evidence suggested that miR-514a-5p functions as a tumor suppressor gene in tumors. Next, we verified that miR-514a-5p directly combined with the 3′-UTR of C22orf46. Hence, we hypothesized that circ-CCAC1 induces the promotion of ACC malignancy by way of its interactions with miR-514a-5p to upregulate C22orf46 expression. Furthermore, a rescue assay demonstrated that the oncogenic role of circ-CCAC1 is partially dependent on its regulation of C22orf46 in ACC cells. C22orf46 was rarely studied before. The functions and mechanisms of C22orf46 are still unclear. To the best of our knowledge, this is the first study revealing the oncogenic role of C22orf46 in human cancers. In addition, we found it could be regulated at the posttranscriptional level in ACC cells. However, there are still some limitations within the study. For example, the detailed downstream targets of C22orf46 are worthy of investigation. The animal study is needed to validate the *in vitro* data. Additionally, a larger cohort of patients should be included in the study to further identify the clinical value of circ-CCAC1 in ACC patients.

To sum up, circ-CCAC1 functions as a sponge for miR-514a-5p to enhance C22orf46 expression, which subsequently contributes to ACC tumorigenesis and progression. These findings may provide additional insights into the molecular events responsible for ACC carcinogenesis.

## Data Availability

The datasets used in the study are available from the corresponding author.

## Supplementary Materials

Supplementary Materials Table S1: clinical details of adrenocortical carcinoma patients.
