# Supplementary material for: Circular RNA circ-CCAC1 Facilitates Adrenocortical Carcinoma Cell Proliferation, Migration, and Invasion through Regulating the miR-514a-5p/C22orf46 Axis
Source: Biomed Res Int. 2020 Oct 26;2020:3501451. doi: 10.1155/2020/3501451 (PMC7641692; doi:10.1155/2020/3501451)
Supplement: Supplementary Materials — Table S1: clinical details of adrenocortical carcinoma patients. [file 3501451.f1.docx]

**Table S1**

| Patient number | OS(1=Dead, 0=Alive) | OS(Months) | circ-CCAC1 expression(1=High, 0=Low) |
| --- | --- | --- | --- |
| 1 | 1 | 6 | 1 |
| 2 | 1 | 6 | 1 |
| 3 | 1 | 6 | 0 |
| 4 | 1 | 8 | 1 |
| 5 | 1 | 8 | 1 |
| 6 | 1 | 9 | 1 |
| 7 | 1 | 9 | 1 |
| 8 | 1 | 11 | 0 |
| 9 | 1 | 12 | 1 |
| 10 | 1 | 12 | 1 |
| 11 | 1 | 14 | 0 |
| 12 | 1 | 16 | 1 |
| 13 | 1 | 18 | 0 |
| 14 | 1 | 20 | 1 |
| 15 | 1 | 22 | 0 |
| 16 | 1 | 28 | 1 |
| 17 | 1 | 33 | 0 |
| 18 | 1 | 35 | 1 |
| 19 | 1 | 36 | 1 |
| 20 | 1 | 42 | 1 |
| 21 | 1 | 52 | 0 |
| 22 | 0 | 60 | 1 |
| 23 | 0 | 60 | 0 |
| 24 | 0 | 60 | 1 |
| 25 | 0 | 60 | 0 |
| 26 | 0 | 60 | 1 |
| 27 | 0 | 60 | 0 |
| 28 | 0 | 60 | 0 |
| 29 | 0 | 60 | 0 |
| 30 | 0 | 60 | 0 |
| 31 | 0 | 60 | 0 |
| 32 | 0 | 60 | 0 |
| 33 | 0 | 60 | 0 |
| 34 | 0 | 60 | 0 |
| 35 | 0 | 60 | 1 |
| 36 | 0 | 60 | 1 |
| 37 | 0 | 60 | 1 |
| 38 | 0 | 60 | 1 |
| 39 | 0 | 60 | 0 |
| 40 | 0 | 60 | 0 |
| 41 | 0 | 60 | 1 |
| 42 | 0 | 60 | 1 |
| 43 | 0 | 60 | 0 |
| 44 | 0 | 60 | 0 |
| 45 | 0 | 60 | 0 |
| 46 | 0 | 60 | 0 |
| 47 | 0 | 60 | 0 |
| 48 | 0 | 60 | 1 |
